# The jingle jangle fallacy in digital well-being research is hindering scientific progress

**DOI:** 10.1186/s44263-026-00327-1

**Published:** 2026-09-16

**Authors:** Christian Montag, Jon D. Elhai

**Affiliations:** 1 Institute of Artificial Intelligence and Brain Sciences, University of Macau, Macau SAR, China; 2 Department of Computer Science, University of Macau, Macau SAR, China; 3 Department of Psychology, University of Macau, Macau SAR, China; 4 Department of Psychology, University of Toledo, Toledo, OH USA; 5 Department of Neurosciences and Psychiatry, University of Toledo, Toledo, OH USA

In this Comment, the ‘jingle jangle fallacy’ in digital well-being studies is discussed. This fallacy around nomenclatures hampers progress in answering precisely what healthy and empowering technology use looks like. We mention the importance of reaching consensus on the blurry concept of digital well-being, hopefully leading to cumulative empirical insights. 

## The jingle jangle fallacy

In academic research stemming from disciplines such as psychology, communication science and neuroscience, there is global discussion about the consequences of digitalization on individual well-being. Among other issues, current discussion focuses on whether overusing digital technologies might harm users in terms of their psychological well-being and underlying neurobiology.

The literature in this field is hard to grasp, because researchers face what is called the “jingle jangle fallacy” [1]. Hanfstingl et al. [2] explained the jingle jangle fallacy as follows: “*Jingle fallacies arise when two or more distinct psychological phenomena are erroneously labeled with the same term*,* while jangle fallacies occur when different terms are used to describe the same phenomenon.*” (p. 1). For instance, one could think of investigating associations between (excessive) technology use and life satisfaction to shed light on the digital well-being complex [3]. Both critical terms in the previous sentence, “excessive technology use” and “life satisfaction”, might be conceptualized in various ways. Regarding the term “excessive technology use,” some people might understand that this term refers to spending much time online, while others might describe it as addictive or compulsive online use. So, researchers could speak of excessive online use (same label) but understand something different when using the term (representing a jingle fallacy). Regarding life satisfaction, the same problems could appear. For instance, different assessment methods for life satisfaction might be chosen, and some researchers might even see the absence of depression also as being a valid well-being measure. Of course, depression and life satisfaction are inversely linked to each other, but they tap very differently into the overall well-being theme (clinical psychology vs. positive psychology – the latter focusing on well-being of persons rather than psychopathology), which is then investigated in the context of digital use variables.

The jangle fallacy could be observed when people assess addictive technology use (hence items with an addiction framework are administered) but use different terminology such as “problematic technology use” or “addictive technology use.” Of note, problematic technology use is very broad and could also mean cyberbullying or other “problematic” online behavior.

In sum, the jingle jangle fallacy causes problems both when researchers operationalize variables in the realm of digital terms, and the same issues arise around operationalizing variables involving the term “well-being.”

## Defining digital well-being

**Fig. 1:**
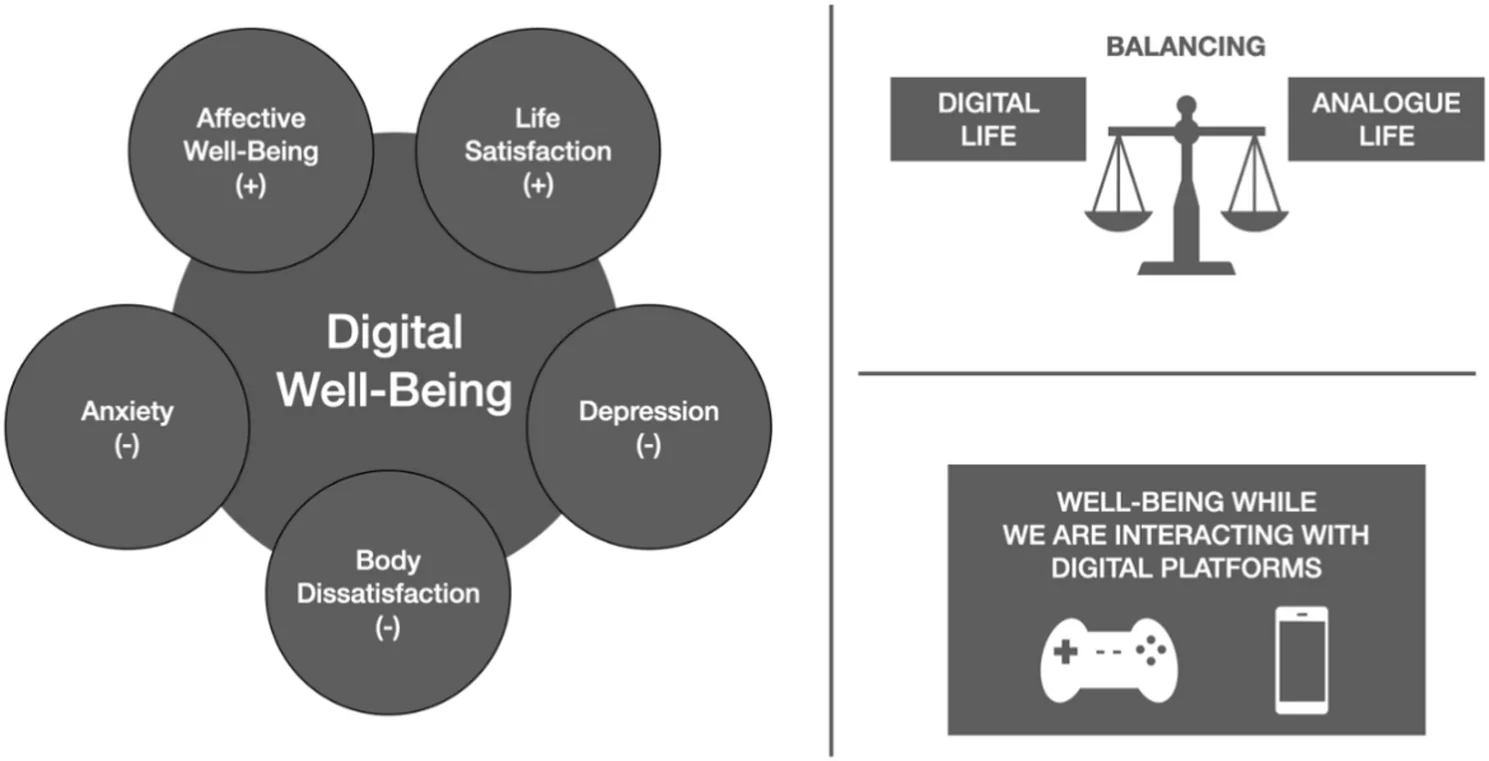
The digital well-being complex: On the left side of Fig. 1, the large area of digital well-being research is described, whereas one’s well-being (often differently operationalized) might be linked to diverse phenotypes, which can be intercorrelated to some extent but are not the same. The right upper and right lower sides depict two different operationalizations of the digital well-being concept. The right upper side depicts the successful balancing of one’s digital and analogue life when speaking of digital well-being. The lower right side focuses on well-being of a person, when interacting with a specific platform

Further complicating a full understanding of relationships between technology use and well-being is the lack of a generally accepted definition of digital well-being. Nevertheless, some important proposals have been made. One prominent definition has been put forward stating that digital well-being can be described by “*a subjective individual experience of optimal balance between the benefits and drawbacks obtained from mobile connectivity*” [4] (p. 932). But what does this optimal balance look like? Can it simply be assessed by the number of hours spent in the more digital vs. more analogue realm? Likely not, because prolonged time spent on social media platforms is only weakly associated with well-being [5]. Such a view might also be dependent on the user’s age group, because for younger users it might be important to reduce technology use to have sufficient time for the fulfillment of important psycho-developmental tasks. Researchers follow the idea that overuse of digital technology itself might negatively impact our overall well-being. Such observations would speak to negative carry-over effects from the digital to analogue life, disturbing our well-being (as Fig. 1 in the upper right half could be interpreted).

There are other definitions of digital well-being which further complicate discussion in the field. For instance, recent work mentioned digital well-being to refer “*to the impact of digital technologies on what it means to live a life that is good for a human being in an information society*” (cited after [6], p. 2313; the original book “The Fourth Revolution” by Luciano Floridi published in Oxford University Press could not be accessed). And Büchi mentions that “*Digital well-being concerns individuals’ subjective well-being in a social environment where digital media are omnipresent.*” [7] (p. 172). Hence, this perspective moves away from the focus of well-being while interacting with a technological device (or being on a digital platform; see lower right half of Fig. 1) but instead investigates the larger picture of people’s well-being in societies where a growing amount of everyday life and human communication is happening in the digital realm.

## Future directions

As mentioned, consensus must be found among scientists regarding the appropriate choice of survey measures to reduce problems around the jingle jangle fallacy. Conceptually, it would be valuable to start finding consensus on how to best measure concepts such as well-being or digital use. As this will not be an easy task, perhaps larger expert panels will be able to rank the relevance of questions tapping more or less into digital well-being. Here one could ask if it is necessary to directly tap into a psychopathological approach to understand digital well-being, or should researchers choose more research-oriented approaches stemming from positive psychology? And when speaking about digital well-being: Do we directly need to tap into the dark side of human nature such as depression or anxiety? Or can we instead answer what kinds of measures could lead to improvement in well-being while still spending some time on a platform or living in digitally connected societies? Of course, it is not forbidden to conduct research on questions dealing with addictive use of technology or on links between social media use and such problems as eating disorders. But perhaps this line of research should be labeled differently (e.g., “digital ill-being”?). This issue also touches on an old dispute, namely if absence of negative affect results in well-being [8]. Issues may also arise when one plans to use established questionnaires. One of the most prominent measures in the assessment of problematic social media use is the Bergen Social Media Addiction Scale [9] consisting of six items using classic symptoms of substance use disorder, but in the context of social media. In the meanwhile, it becomes clear that some of the symptoms translated to social media use are not related with psychopathology [10], hence administering this questionnaire as is might lead to over pathologizing everyday life behavior.

The issues pointed out could be further spelled out in more detail by tackling the following questions, which can also help to set up a structured research program. The following questions could be a guiding light for the assessment of technology use: Is the time or addictive behavior on a device/a group of applications/a specific application measured? Do we speak of active or passive use of technology, for instance of more social activity vs. passive use of technology? For the well-being complex, the questions could be: Do we speak of life satisfaction/emotional well-being/negative emotions/depressed states or even full-blown psychopathology?

## Conclusions

The digital well-being complex suffers from the jingle jangle fallacy, and in the absence of a unifying definition and theory, progress will be difficult. Science makes progress when studies can build on each other, allowing cumulative scientific insights. Only by considering the different operationalization of the digital well-being complex and perhaps by also bringing together a much-needed expert panel aiming to find consensus, can scientific progress in this field be accelerated.

## Data Availability

No datasets were generated or analysed during the current study.

## References

[1] Kross E, Verduyn P, Sheppes G, Costello CK, Jonides J, Ybarra O. Social Media and Well-Being: Pitfalls, Progress, and Next Steps. Trends in Cognitive Sciences [Internet]. 2021 [cited 2022 Jan 22];25:55–66. 10.1016/j.tics.2020.10.005 33187873

[2] Hanfstingl B, Oberleiter S, Pietschnig J, Tran US, Voracek M. Detecting jingle and jangle fallacies by identifying consistencies and variabilities in study specifications – a call for research. Front Psychol [Internet] Front. 2024. 10.3389/fpsyg.2024.1404060. [cited 2024 Dec 14];15. PMC11393684 39282677

[3] Lachmann B, Sariyska R, Kannen C, Cooper A, Montag C. Life satisfaction and problematic Internet use: Evidence for gender specific effects. Psychiatry Res [Internet]. 2016;238:363–7. 10.1016/j.psychres.2016.02.017. [cited 2021 Jan 23];. 27011335

[4] Vanden Abeele MMP. Digital Wellbeing as a Dynamic Construct. Communication Theory [Internet]. 2021 [cited 2023 May 17];31:932–55. 10.1093/ct/qtaa024

[5] Huang C. Time Spent on Social Network Sites and Psychological Well-Being: A Meta-Analysis. Cyberpsychology, Behavior, and Social Networking [Internet]. 2017 [cited 2022 Dec 19];346–54. https://www.researchgate.net/publication/317628925_Time_Spent_on_Social_Network_Sites_and_Psychological_Well-Being_A_Meta-Analysis. Accessed 19 Dec 2022. 10.1089/cyber.2016.0758 28622031

[6] Burr C, Taddeo M, Floridi L. The Ethics of Digital Well-Being: A Thematic Review. Sci Eng Ethics. 2020;26:2313–43. 10.1007/s11948-020-00175-8. 31933119 PMC7417400

[7] Büchi M. Digital well-being theory and research. New Media & Society. SAGE Publications. 2024;26:172–89. 10.1177/14614448211056851.

[8] Davis KL, Montag C. Jaak Panksepp’s Primary Emotions Associate with Diener’s Global Life Satisfaction: How Low Arousal of the SADNESS/Separation Distress Could Form the Core of Life Satisfaction? Personality Neurosci. 2024;7:e11. 10.1017/pen.2024.4 PMC11706680 39776538

[9] Andreassen CS, Billieux J, Griffiths MD, Kuss DJ, Demetrovics Z, Mazzoni E, et al. The relationship between addictive use of social media and video games and symptoms of psychiatric disorders: A large-scale cross-sectional study. Psychology of Addictive Behaviors. Volume 30. US: American Psychological Association; 2016. pp. 252–62. 10.1037/adb0000160. 26999354

[10] Fournier L, Schimmenti A, Musetti A, Boursier V, Flayelle M, Cataldo I, et al. Deconstructing the components model of addiction: an illustration through addictive use of social media. Addict Behav. 2023;143:107694. 10.1016/j.addbeh.2023.107694. 36940658

